# Macrophage‐to‐Myofibroblast Transdifferentiation Contributes to Pulmonary Fibrosis via the MERTK‐SPP1‐SRC‐TKS5 Signaling Axis

**DOI:** 10.1002/advs.75620

**Published:** 2026-05-14

**Authors:** Yungeng Wei, Hua Guo, Xiangsheng Yang, Xiao Xiao Tang

**Affiliations:** ^1^ State Key Laboratory of Respiratory Disease National Clinical Research Center for Respiratory Disease National Center for Respiratory Medicine Guangzhou Institute of Respiratory Health The First Affiliated Hospital of Guangzhou Medical University Guangzhou China; ^2^ Guangzhou National Laboratory Guangzhou China

**Keywords:** idiopathic pulmonary fibrosis, macrophage, macrophage‐to‐myofibroblast transdifferentiation (MMT), MERTK, TKS5

## Abstract

In recent years, macrophage‐to‐myofibroblast transdifferentiation (MMT) has been found in fibrosis‐related diseases. We confirmed the presence of MMT in human idiopathic pulmonary fibrosis (IPF) lungs and bleomycin‐induced murine model using immunological and molecular methods. Mechanistically, ligand (GAS6)‐mediated activation of MERTK on macrophages initiates a sequential signaling cascade involving SPP1, SRC, and TKS5, which collectively drives the transdifferentiation program. Conversely, knockout of MERTK specifically in macrophages or adeno‐associated virus (AAV)‐mediated knockdown of TKS5 effectively disrupted this MERTK‐SPP1‐SRC‐TKS5 axis, potently suppressing MMT in vitro and in vivo. These interventions significantly attenuated the progression of pulmonary fibrosis, as evidenced by comprehensive assessments including microCT, pulmonary function test, and histopathological analysis. Our findings establish MMT as a key pathogenic mechanism and identify the MERTK‐initiated signaling axis as a novel therapeutic target.

## Graphical abstract

MERTK is upregulated in fibrotic macrophages and regulates the expression and activity of SRC and TKS5 through SPP1, mediating transdifferentiation of macrophages‐to‐myofibroblasts (MMT) and promoting pulmonary fibrosis. The figure was created with BioRender.com.

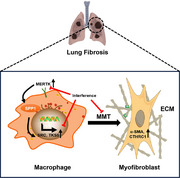

## 1 Introduction

Idiopathic pulmonary fibrosis (IPF) is a relentlessly progressive lung disease with a median survival of 2–3 years post‐diagnosis, characterized by aberrant extracellular matrix (ECM) deposition and dysfunctional alveolar repair [1, 2, 3]. While the role of epithelial injury and fibroblast activation is well‐established, emerging evidence implicates that macrophages are becoming pivotal orchestrators of fibrogenesis through dynamic phenotypic switching and crosstalk with stromal cells [4, 5]. Macrophages in IPF exhibit a paradoxical duality: they initiate tissue repair but then drive fibrosis through unresolved inflammation and direct ECM reprogramming. This functional plasticity of macrophages makes them a potential therapeutic target, but the molecular mechanisms by which they promote fibrotic reprogramming remain elusive.

The TAM (Tyro3 [TYRO3 protein tyrosine kinase 3], Axl [anexelekto], and MERTK [MER proto‐oncogene, tyrosine kinase]) family receptor tyrosine kinase MERTK, expressed predominantly in macrophages, is classically recognized for its role in efferocytosis and inflammation resolution [6, 7]. Our prior work revealed that MERTK regulates efferocytosis and exacerbates IPF by promoting profibrotic cytokine secretion. [8] A previous single‐cell RNA sequencing study revealed that MERTK^+^SPP1^+^ macrophages in IPF lungs co‐express mesenchymal markers (e.g., α‐SMA), indicating an involvement of MERTK in myofibroblast differentiation – a phenomenon termed macrophage‐to‐myofibroblast transdifferentiation (MMT) [9]. However, this study described cell identity and spatial association but did not establish whether MERTK/SPP1^+^ macrophages undergo transdifferentiation into myofibroblasts or define the downstream signaling mediating such a fate change. Intriguingly, TAM receptors have been implicated in epithelial‐mesenchymal transition (EMT) [10, 11], yet their role in macrophage plasticity during fibrosis remains unexplored.

Secreted phosphoprotein 1 (SPP1, osteopontin), a multifunctional glycoprotein upregulated in IPF, drives macrophage polarization toward a pro‐fibrotic M2 phenotype and potentiates TGF‐β signaling. [12] It is reported that MERTK signaling acts as an upstream regulator of SPP1 to regulate the M2 polarization of macrophages. [13] Emerging single‐cell transcriptomic studies have unveiled a spectrum of macrophage subsets in IPF lungs, including a unique MERTK^+^SPP1^+^ subpopulation that co‐expresses both pro‐fibrotic markers and mesenchymal genes. [9] Notably, these transitional macrophages localize preferentially at the fibroblastic foci – epicenters of ECM deposition – and exhibit enhanced migratory capacity. Such spatial and functional specialization implies that macrophage plasticity is not merely a bystander but instead an active contributor to fibrotic niche formation. MERTK‐driven SPP1 upregulation also serves as a critical molecular bridge linking receptor tyrosine kinase signaling to downstream cytoskeletal remodeling. SPP1 recruits and activates SRC kinase [14], thereby directly phosphorylating TKS5 (Tyr kinase substrate with five SH3 domains) [15, 16], a scaffold protein essential for podosome assembly, which is crucial for matrix degradation and mesenchymal transition. In cancer, SRC‐TKS5 signaling facilitates tumor cell invasion by degrading basement membranes [17, 18, 19]; however, its relevance to macrophage‐driven fibrosis remains unknown. Thus, while prior work linked MERTK and SPP1 expression to a profibrotic macrophage phenotype, the mechanistic chain connecting receptor activation to cytoskeletal remodeling and cellular fate change in fibrosis remained unexplored. Here we test the hypothesis that MERTK activation induces SPP1‐dependent SRC activation and subsequent TKS5 phosphorylation to drive podosome formation and MMT, providing mechanistic and causal evidence.

In summary, beyond prior descriptive identification of MERTK^+^SPP1^+^ macrophage population in IPF, our study provides the first mechanistic and causal evidence that macrophage‑intrinsic MERTK signaling drives MMT via an SPP1‐SRC‐TKS5 cascade and that interruption of this axis prevents MMT and attenuates pulmonary fibrosis in vivo. By integrating multimodal approaches, we provide conclusive evidence that MMT is a mechanistically distinct pathway linking macrophage plasticity to ECM remodeling in IPF. These findings position macrophages as active engagers in the fibrotic niche, rather than passive cytokine producers. Importantly, our identification of MERTK as a druggable node in MMT opens new avenues for combination therapies that may complement existing anti‐fibrotics by targeting MMT – a strategy with potential to halt disease progression rather than merely slow functional decline.

## 2 Results

### 2.1 Macrophage‐to‐Myofibroblast Transdifferentiation (MMT) is Involved in Pulmonary Fibrosis

**FIGURE 1:**
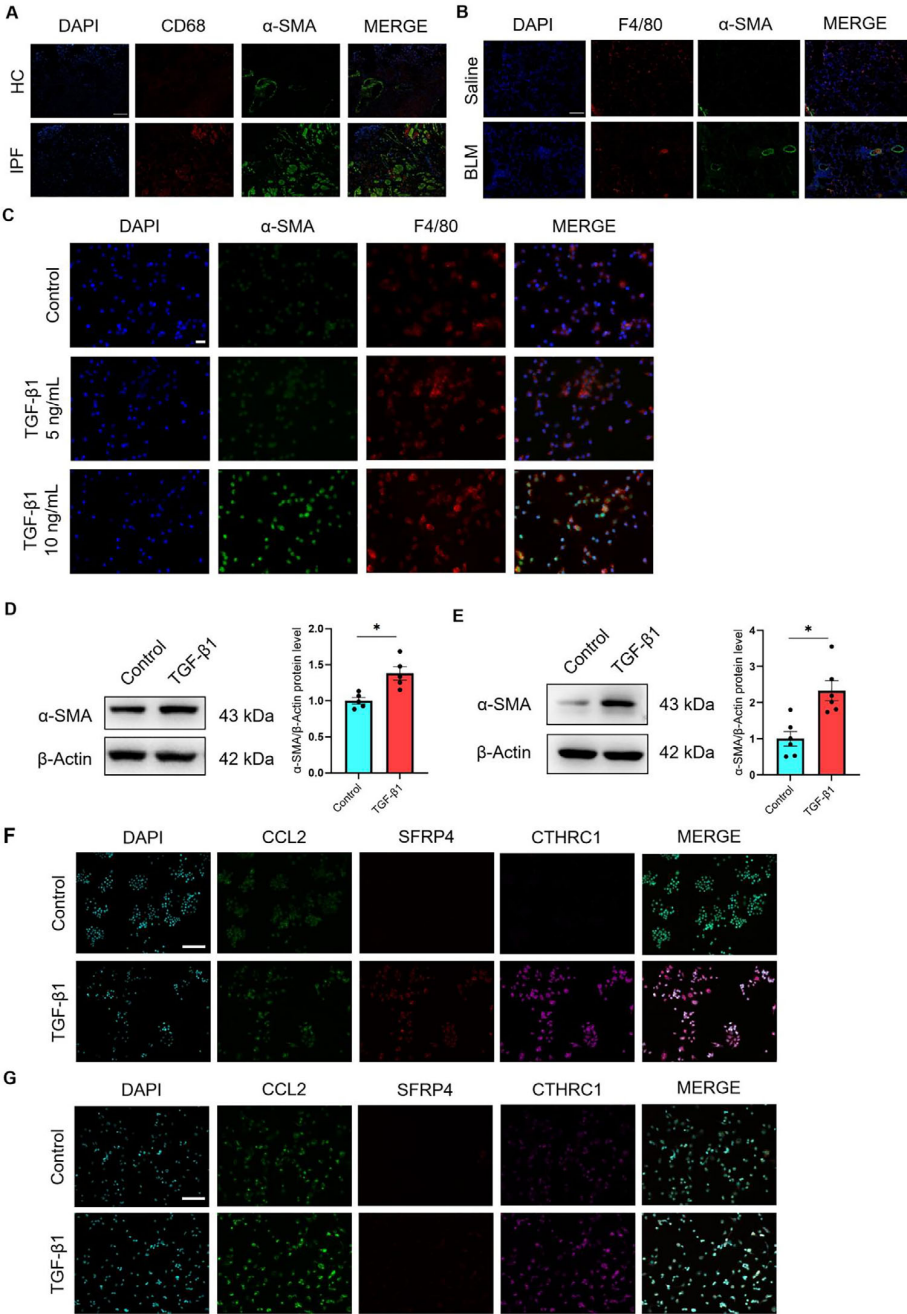
Macrophage‐to‐myofibroblast transdifferentiation (MMT) is involved in pulmonary fibrosis. (A) Immunofluorescence staining detects co‐localization of the fibroblast marker α‐SMA with the macrophage marker CD68 in lung tissues of IPF patients. HC, Health Control; IPF, Idiopathic pulmonary fibrosis. Scale bar = 100 µm. *n* = 5. (B) Immunofluorescence staining detects co‐localization of fibroblast marker α‐SMA with macrophage marker F4/80 in lung tissues of mice with bleomycin (BLM)‐induced pulmonary fibrosis. Scale bar = 100 µm. *n* = 5. (C) Immunofluorescence staining detects MMT in MH‐S cells induced by TGF‐β1 stimulation for 48 h. Scale bar = 20 µm. *n* = 5. (D) MH‐S cells were treated with TGF‐β1 for 48 h, and WB assayed for α‐SMA expression. *n* = 5. (E) THP‐1 cells were induced to differentiate into macrophages by PMA and treated with TGF‐β1 for 48 h. Expression of α‐SMA was detected by WB. *n* = 6. (F) Immunofluorescence staining to detect the expression of CCL2, SFRP4, and CTHRC1 in MH‐S cells treated with TGF‐β1. Scale bar = 100 µm. (G) Immunofluorescence staining to detect the expression of CCL2, SFRP4, and CTHRC1 in PMA‐pretreated THP‐1 cells stimulated with TGF‐β1. Scale bar = 100 µm. The data were assessed by an unpaired two‐sided Student's *t*‐test and are shown as mean ± SEM. ^*^ *p* < 0.05.

Fibroblasts are one of the major effector cells in pulmonary fibrosis, and their aberrant activation into myofibroblasts in pulmonary fibrosis leads to an abnormal increase in the extracellular matrix. Myofibroblasts in fibrotic diseases originate from various sources, including macrophages, a process known as macrophage‐myofibroblast transdifferentiation. To investigate whether MMT exists in idiopathic pulmonary fibrosis, we first examined the co‐localization of α‐SMA (a marker of myofibroblasts) with CD68 (a marker of macrophages) in lung tissue sections from IPF patients using immunofluorescence staining, and found that, compared with lung tissues from healthy controls, not only was the expression of α‐SMA increased in lung tissues from IPF patients, but also there was a significant co‐localization of α‐SMA with CD68 (Figure 1A). This result was also replicated in bleomycin (BLM)‐induced pulmonary fibrosis mice (Figure 1B). It has been demonstrated that M2‐polarized macrophages are more susceptible to MMT, here we performed immunofluorescence staining of lung tissue sections from BLM mice and found that macrophages in BLM‐instilled mice underwent M2‐polarization in lung tissues (Figure S1A), and that there was co‐localization of α‐SMA with the macrophage M2‐polarization marker CD206 (Figure S1B), which is consistent with the fact that the macrophages that undergo MMT are predominantly M2‐polarized macrophages. Next, to make it clear that macrophages are capable of undergoing MMT, we used two cell lines for in vitro experiments. We administered a pro‐fibrotic cytokine TGF‐β1 to human‐derived macrophages and mouse‐derived macrophages, respectively, and consistent with the results of the in vivo experiments, increased expression of α‐SMA was observed in both types of cells (Figure 1D,E). In addition, we isolated mouse bone marrow cells and induced them differentiation into macrophages (BMDMs), following stimulation with TGF‐β1, the expression of α‐SMA in BMDMs significantly increased (Figure S2A,B); We also isolated alveolar macrophages (AMs) from bronchoalveolar lavage fluid (BALF) of BLM‐induced mice and found that AMs from the BLM group expressed higher levels of α‐SMA compared to those from the Saline group (Figure S2C). These results demonstrate that MMT can also occur in primary cultured macrophages. Recent studies have shown that fibroblasts in fibrotic lung tissues can be divided into inflammatory fibroblasts (mainly expressing CCL2 and SFRP4) and pro‐fibrotic fibroblasts (CTHRC1 as a marker) [20]. To determine what type of fibroblasts the macrophages are converted to following MMT, we examined the expression of these markers using immunofluorescence staining and found remarkable CTHRC1 expression in macrophages after TGF‐β1 treatment (Figure 1F,G). These results suggest that macrophages have the potential to transdifferentiate into myofibroblasts with pro‐fibrotic capacity. In addition, TGF‐β1‐treated macrophages exhibit a slender, long, striped morphology, similar to that of myofibroblasts (Figure S1C). Overall, these results demonstrate that macrophages can transdifferentiate to myofibroblasts and participate in the process of pulmonary fibrosis.

### 2.2 The Macrophages Undergoing MMT are Predominantly M2 Phenotype

**FIGURE 2:**
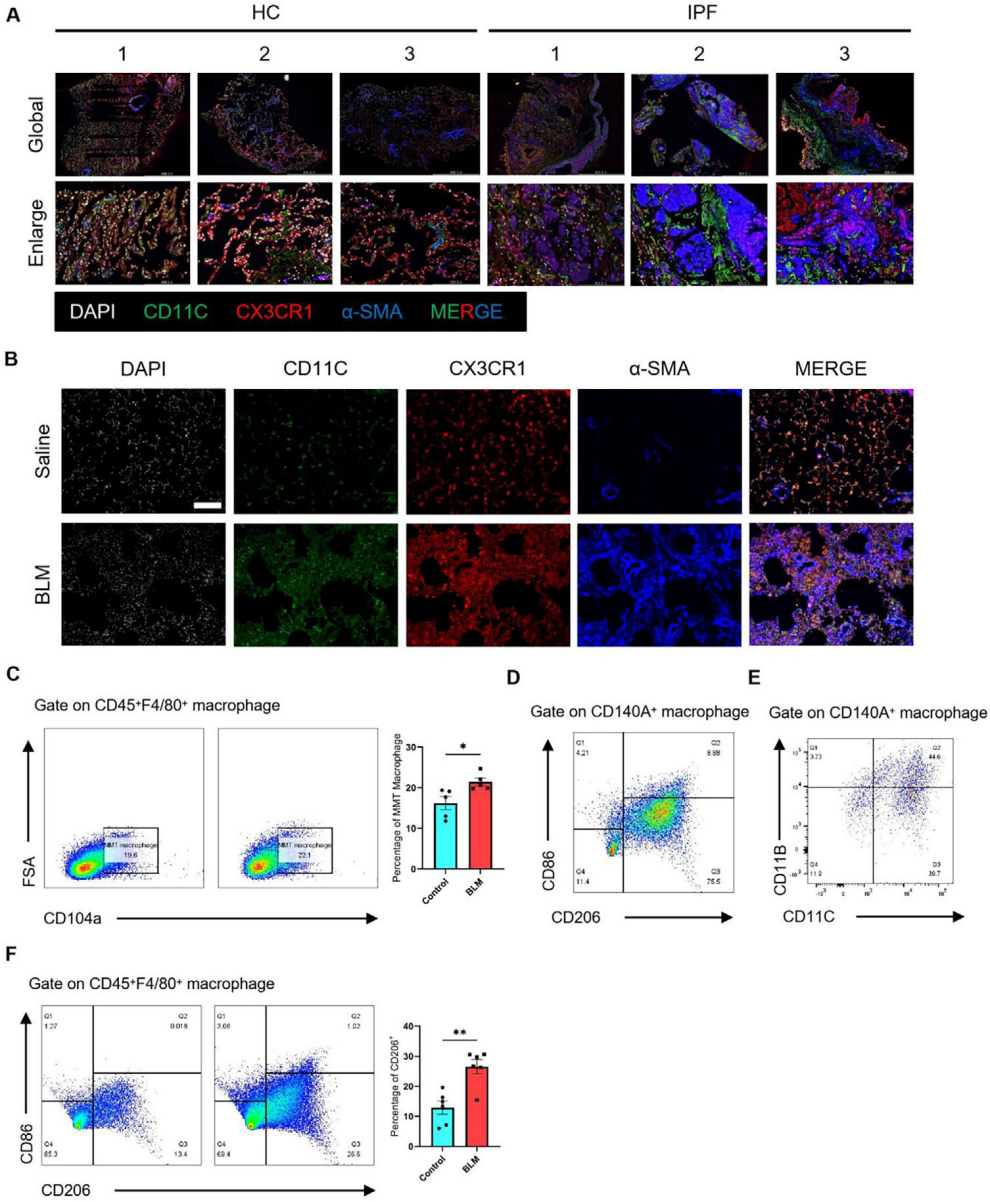
MMT occurrence in different subpopulations of macrophages. (A) Immunofluorescence staining detects co‐localization of recruitment‐derived macrophages (CX3CR1, red), tissue‐resident alveolar macrophages (CD11C, green), and fibroblasts (α‐SMA, blue) in lung tissue sections from patients with IPF. HC, Health Control; IPF, Idiopathic pulmonary fibrosis. *n* = 3. (B) Co‐localization of fibroblast marker α‐SMA with recruitment‐derived macrophages (CX3CR1^+^) and tissue‐resident alveolar macrophages (CD11C^+^) in lung tissues of mice with BLM‐induced pulmonary fibrosis detected by immunofluorescence staining. *n* = 3, Scale bar = 100 µm. (C) Flow cytometry detection of the proportion of macrophages undergoing MMT in BLM‐induced mouse lung tissue, *n* = 5. (D) Ratio of CD86 to CD206 in macrophages undergoing MMT detected by flow cytometry. (E) Proportion of interstitial and alveolar macrophages undergoing MMT detected by flow cytometry. (F) Flow cytometry detection of CD86 and CD206 percentages in lung tissue macrophages from BLM‐instilled mice, *n* = 6. The data were assessed by an unpaired two‐sided Student's *t*‐test and are shown as mean ± SEM. ^*^ *p* < 0.05, ^**^ *p* < 0.01.

Lung macrophages consist of two main subsets: tissue‐resident alveolar macrophages and interstitial macrophages. To investigate which type of macrophages undergo MMT in pulmonary fibrosis, we first performed immunofluorescence staining using lung tissue sections from IPF patients and healthy controls, and found that α‐SMA co‐localized more with the marker of interstitial macrophages – CX3CR1, and less with the marker of tissue‐resident alveolar macrophages – CD11C (Figure 2A). However, immunofluorescence staining of BLM‐induced pulmonary fibrosis in mice revealed that although MMT occurs predominantly in interstitial macrophages, tissue‐resident alveolar macrophages are also capable of MMT (Figure 2B). We next examined the proportion of macrophages expressing fibroblast marker CD140A in lung tissue macrophages from mice with BLM‐induced pulmonary fibrosis using flow cytometry and found that BLM increased the proportion of CD140A^+^ macrophages, indicating an increased number of macrophages undergoing MMT in the BLM group (Figure 2C). At the same time, we analyzed the proportion of CD86 and CD206 in macrophages that underwent MMT and found that macrophages that underwent MMT were predominantly CD206^+^ (Figure 2D). We used flow cytometry to analyze the proportions of interstitial macrophages and alveolar macrophages to macrophages with MMT, and the results showed that MMT can occur in both interstitial macrophages and tissue‐resident alveolar macrophages (Figure 2E). Our flow cytometry results illustrated that macrophages from BLM‐induced pulmonary fibrosis mice predominantly undergo M2 polarization (Figure 2F). The above results suggest that macrophages undergo MMT during fibrosis, and the macrophages that undergo MMT is predominantly M2‐type.

### 2.3 MERTK^+^SPP1^+^ Macrophages Undergo MMT

**FIGURE 3:**
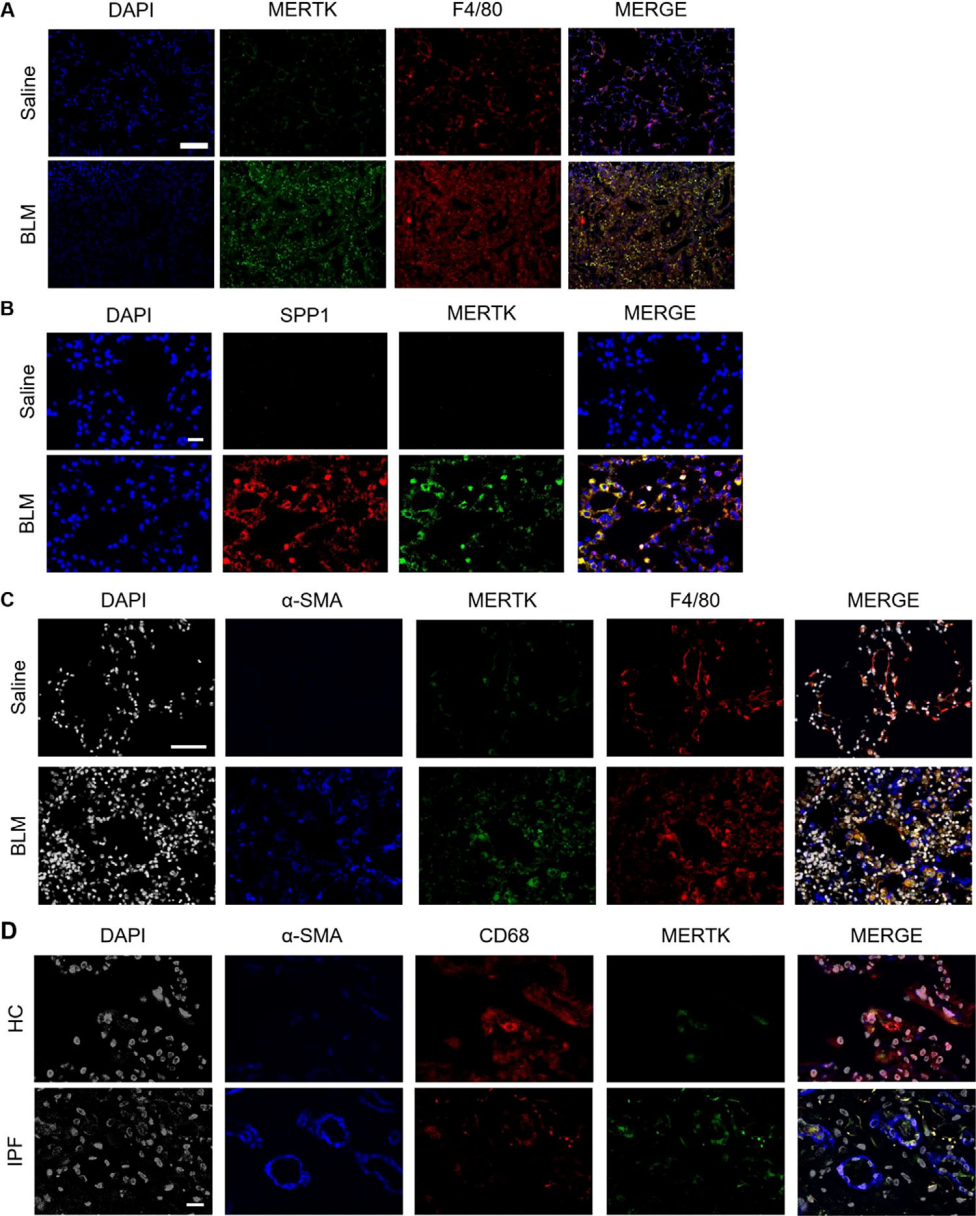
Macrophages undergoing MMT highly express MERTK. (A) Detection of macrophage MERTK expression in BLM‐induced mouse lung tissue sections by immunofluorescence staining. Scale bar = 100 µm. (B) Expression and co‐localization of MERTK and SPP1 in lung sections from BLM‐induced mouse detected by immunofluorescence staining. Scale bar = 100 µm. (C) Immunofluorescence staining to detect co‐localization of F4/80, MERTK, and α‐SMA in lung tissues of BLM mice, scale bar = 20 µm. (D) Immunofluorescence staining to detect co‐localization of CD68, MERTK, and α‐SMA in lung tissues of IPF patients, scale bar = 20 µm.

Our previous study found that MERTK‐dependent efferocytosis in macrophages could promote lung fibrosis by regulating macrophage secretion of pro‐fibrotic factors [8]; however, the inherent pro‐fibrotic mechanism of MERTK remains to be investigated. Here, we speculate that MERTK may be involved in MMT in macrophages. We first found increased MERTK expression of macrophages by immunofluorescence staining (Figure 3A). It has been found that there is a population of SPP1^+^ macrophages in the lung tissue of IPF patients, which are highly active in pulmonary fibrosis and highly express MERTK [9]. We further examined the co‐localization of MERTK and SPP1 in lung sections from BLM‐induced mice and found that they were highly co‐localized (Figure 3B). Accordingly, we hypothesized that MERTK^+^SPP1^+^ macrophages were involved in the process of MMT. Our immunofluorescence staining results revealed an increase in MERTK expression in macrophages that underwent MMT (Figure 3C,D). Meanwhile, TGF‐β1 treatment induced MMT in macrophages, and enhanced the expression of SPP1 and MERTK (Figure S3), consistent with our conjecture that MERTK^+^SPP1^+^ macrophages may be the major contributors to MMT.

### 2.4 MERTK Mediates MMT to Promote Pulmonary Fibrosis

**FIGURE 4:**
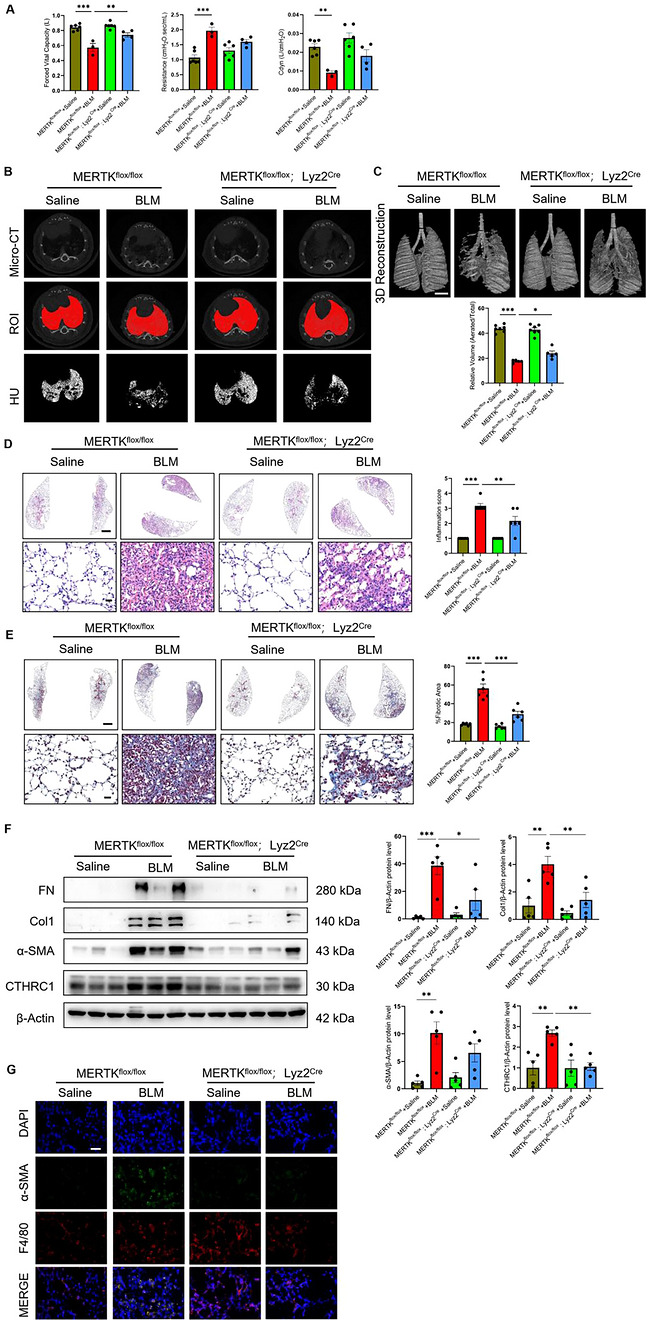
Knockout of MERTK attenuates BLM‐induced pulmonary fibrosis by reducing MMT. (A) Pulmonary function test of mice in each group, *n* = 3–6. (B,C) MicroCT detects lung tissue damage and quantifies the proportion of normally aerated area in each group of mice, *n* = 5–7. (D) HE staining was used to detect and score the degree of inflammatory infiltration in the lung tissue of mice in each group, scale bar = 1 mm (top) and 50 µm (bottom), *n* = 6. (E) Masson staining was used to detect the degree of collagen deposition in the lung tissues of mice in each group, and the percentage of fibrosis was quantified, scale bar = 1 mm (top) and 50 µm (bottom), *n* = 6. (F) Expression of fibrosis‐related factors in the lung tissue of mice in each group was detected by WB, *n* = 5. (G) Co‐localization of α‐SMA and F4/80 in the lungs of each group of mice detected by immunofluorescence staining, scale bar = 50 µm. The data were assessed by one‐way ANOVA and are shown as mean ± SEM. ^*^ *p* < 0.05, ^**^ *p* < 0.01, ^***^ *p* < 0.001.

To investigate whether MERTK plays an important role in MMT, we constructed mice with macrophage‐specific knockout of MERTK (Figure S4) and modeled pulmonary fibrosis by intratracheally instilled of BLM. We first tested pulmonary function in mice and found that macrophage‐specific knockout of MERTK ameliorated BLM‐induced pulmonary impairment (Figure 4A). We then assessed the lung tissue damage using microCT, and found obvious lung tissue damage and reduced proportion of normally aerated lung tissue in BLM‐induced mice; whereas specific knockout of MERTK alleviated the lung tissue damage and enhanced the proportion of normally aerated lung tissue (Figure 4B,C). Next, we examined inflammatory infiltration and the degree of fibrosis in mouse lung tissues by HE staining and Masson staining, and found reduced inflammatory infiltration in mouse lung tissues (Figure 4D) and alleviated fibrosis (Figure 4E) after specific knockout of MERTK. Also, we examined the expression of fibrosis‐associated factors at the protein level, including CTHRC1, FN, COL1, and α‐SMA, and found that specific knockout of MERTK markedly reduced their expression (Figure 4F). Finally, we used immunofluorescence staining to examine the occurrence of MMT in the lung tissues of mice in each group, and found decreased co‐localization of the macrophage marker F4/80 with the marker α‐SMA in myofibroblasts after knockout of MERTK, suggesting that there was a reduction in MMT (Figure 4G). These results suggest that knockout of MERTK in macrophages attenuates BLM‐induced pulmonary fibrosis by decreasing MMT.

### 2.5 Regulation of MMT by MERTK‐SPP1 Signaling

**FIGURE 5:**
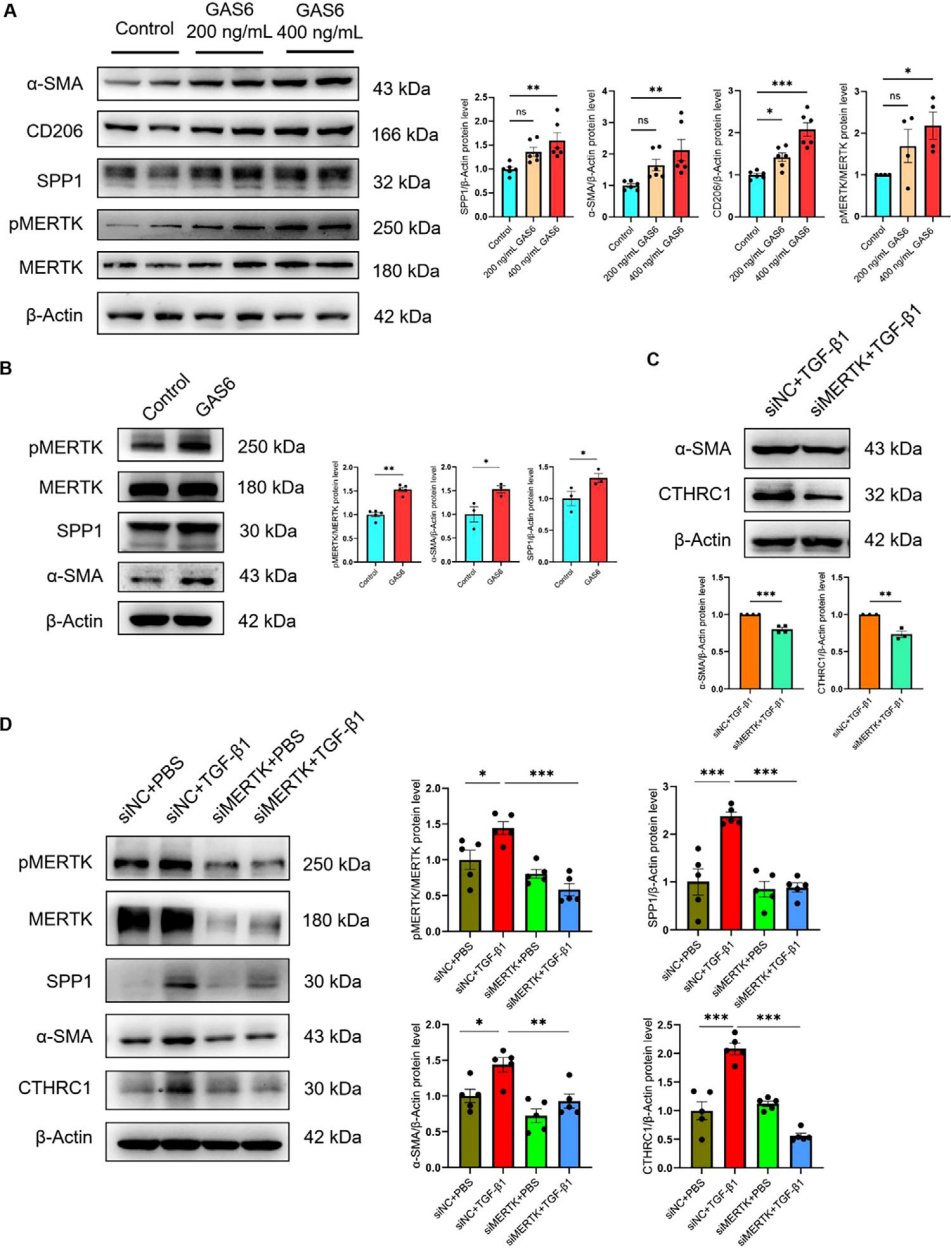
MMT is regulated by MERTK. (A) MH‐S cells were treated with 200 and 400 ng/mL GAS6 for 48 h. Proteins were extracted for WB detection of CD206, SPP1, α‐SMA, MERTK, and pMERTK, *n* = 4–6. (B) THP‐1 cells were induced to differentiate into macrophages by PMA and treated with 400 ng/mL GAS6. Phosphorylation of MERTK and expression of SPP1, α‐SMA were detected by WB, *n* = 3–5. (C) MH‐S cells were transfected with siRNA knockdown of MERTK and then treated with TGF‐β1 for 48 h. Proteins were extracted for WB detection of α‐SMA and CTHRC1, *n* = 3–4. (D) THP‐1 cells were induced to differentiate into macrophages by PMA, transfected with siRNA to knock down MERTK. Phosphorylation of MERTK and expression of SPP1, α‐SMA, and CTHRC1 were detected by WB, *n* = 5. The data in B and C were assessed by an unpaired two‐sided Student's *t*‐test and are shown as mean ± SEM. The data in A and D were assessed by one‐way ANOVA and are shown as mean ± SEM. ns means not significant, ^*^ *p* < 0.05, ^**^ *p* < 0.01, ^***^ *p* < 0.001.

To confirm the role of MERTK in MMT, we treated mouse‐derived macrophages (MH‐S cells) with MERTK ligand GAS6 and found that activating MERTK by GAS6 could upregulate SPP1 expression and promote the occurrence of MMT (Figure 5A). Consistent with this, activation of MERTK in THP‐1‐derived macrophages can also upregulate SPP1 and promote MMT (Figure 5B). Knocking down MERTK reduced the occurrence of MMT in macrophages (Figure 5C,D). These results demonstrated that activation of MERTK induces MMT, while knocking down MERTK reduces TGF‐β1‐induced MMT, thereby confirming the key role of MERTK in MMT.

### 2.6 MERTK^+^SPP1^+^ Macrophages Mediate MMT via the SRC‐TKS5 Signaling Pathway

**FIGURE 6:**
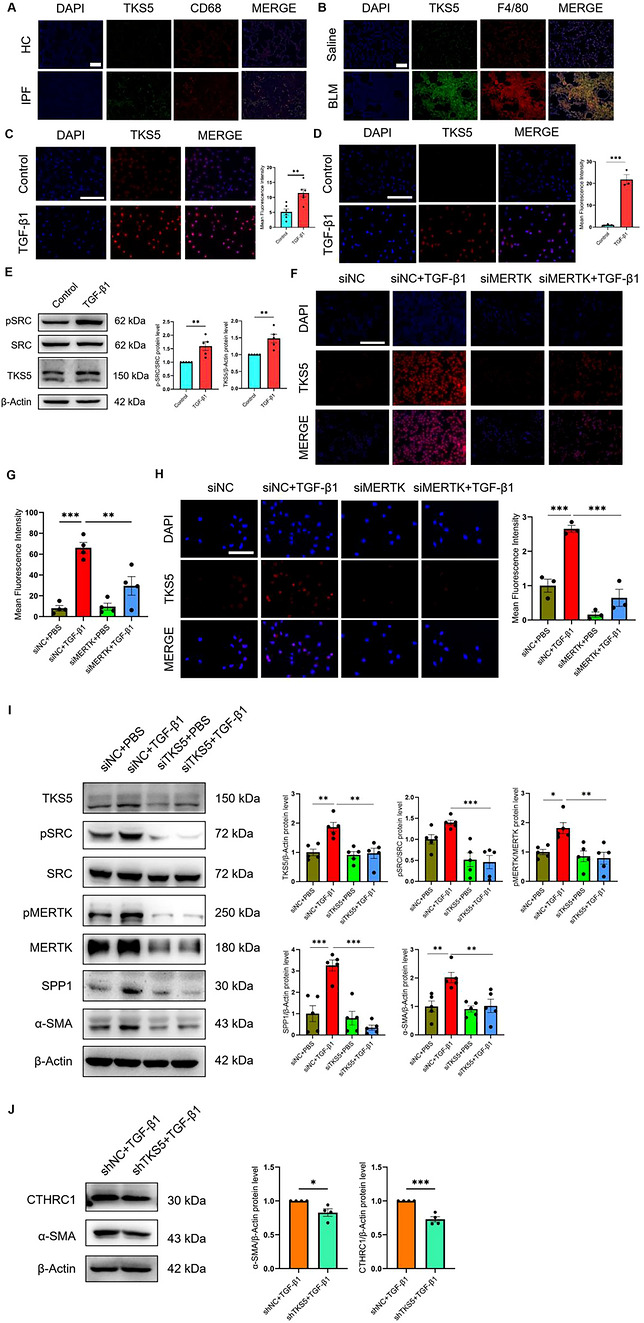
MERTK regulates MMT via the SRC‐TKS5 signaling axis. (A) Immunofluorescence staining for TKS5 (green) expression in macrophages (CD68, red) of lung tissues from IPF patients. Scale bar = 100 µm, *n* = 5–6. (B) Immunofluorescence staining for TKS5 (green) expression in macrophages (F4/80, red) in BLM‐instilled mouse lung tissue sections. Scale bar = 100 µm, *n* = 6. (C) MH‐S cells were treated with TGF‐β1 for 48 h, and TKS5 expression was detected by immunofluorescence staining. Scale bar = 100 µm, *n* = 6. (D) THP‐1 cells were differentiated into macrophages using PMA and then treated with TGF‐β1 for 48 h, and TKS5 expression was detected by immunofluorescence staining. Scale bar = 100 µm, *n* = 3. (E) MH‐S cells were treated with TGF‐β1 for 48 h, and SRC, pSRC, and TKS5 expression were detected by WB, *n* = 5. (F,G) MH‐S cells were transfected with siRNA knockdown of MERTK and then treated with TGF‐β1 for 48 h. Immunofluorescence staining was used to detect the expression of TKS5. Scale bar = 100 µm, *n* = 4. (H) THP‐1 cells were induced to differentiate into macrophages using PMA, transfected with siRNA to knock down MERTK, and then stimulated with TGF‐β1 for 48 h. Immunofluorescence staining was used to detect the expression of TKS5. Scale bar = 100 µm, *n* = 3. (I) THP‐1 was induced to differentiate into macrophages with PMA, transfected with siRNA to knock down TKS5, and then stimulated with TGF‐β1 for 48 h. The expression of MERTK, SRC, pMERTK, pSRC, TKS5, and α‐SMA was detected by WB, *n* = 5. (J) MH‐S cells were transfected with shRNA knockdown of TKS5 and then treated with TGF‐β1 for 48 h. The expression of α‐SMA and CTHRC1 was detected by WB, *n* = 4. The data in C–E and J were assessed by an unpaired two‐sided Student's *t*‐test and are shown as mean ± SEM. The data in G–I were assessed by one‐way ANOVA and are shown as mean ± SEM. ^*^ *p* < 0.05, ^**^ *p* < 0.01, ^***^ *p* < 0.001.

Previous studies have shown that SPP1 can activate TKS5 via activating SRC kinase [14, 16], and TKS5 activity can regulate the migratory capacity of macrophages. [15] Therefore, we hypothesized that MERTK^+^SPP1^+^ macrophages may regulate MMT through the SRC‐TKS5 signaling pathway. Immunofluorescence staining was employed to evaluate TKS5 expression in the lungs of IPF patients and BLM‐instilled mice, as well as in TGF‐β1‐treated macrophages. Upregulation of TKS5 was observed in fibrotic macrophages (Figure 6A–D). Western blot analysis corroborated these results by revealing augmented SRC activation and TKS5 expression in TGF‐β1‐treated macrophages (Figure 6E). However, knockdown of MERTK suppressed TGF‐β1‐induced increase in TKS5 expression (Figure 6F–H). Further, we found that knocking down TKS5 expression in macrophages reduced TGF‐β1‐induced MMT (Figure 6I,J). These data demonstrate that MERTK^+^SPP1^+^ macrophages may regulate MMT through the SRC‐TKS5 signaling pathway.

### 2.7 Effect of In Vivo Knockout of MERTK on the SRC‐TKS5 Signaling

**FIGURE 7:**
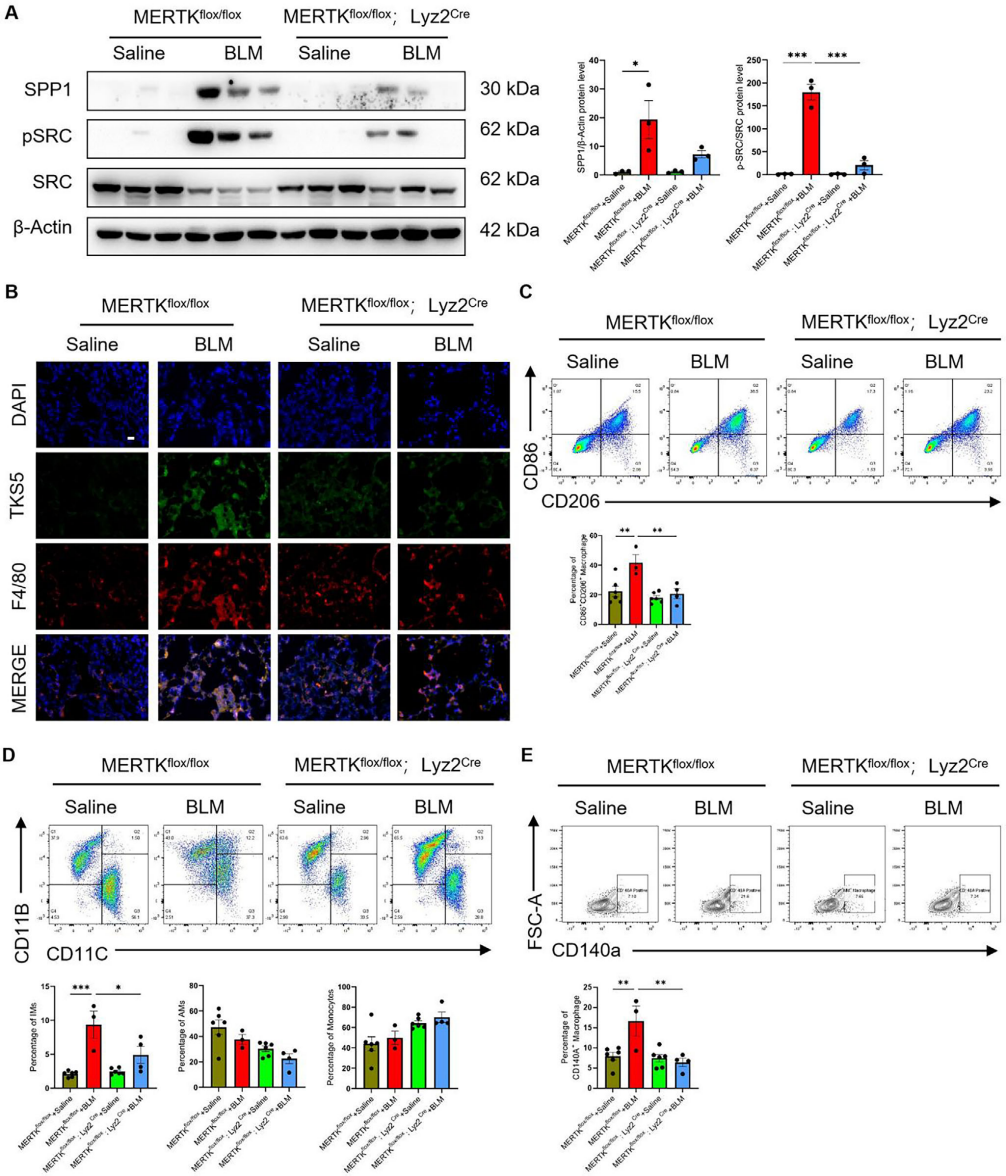
In vivo knockout of MERTK regulates macrophage function through the SRC‐TKS5 signaling axis. (A) WB detected SRC activity and SPP1 expression in each group of mice, *n* = 3. (B) Immunofluorescence staining to detect the expression of TKS5 in macrophages of each group of mice. (C) Detection of M1/M2 polarization of mouse lung macrophages in each group by flow cytometry, *n* = 3–6. (D) The percentage of macrophages of each subpopulation of the mouse lung in each group detected by flow cytometry, *n* = 3–6. (E) The percentage of mouse lung macrophages that underwent MMT in each group detected by flow cytometry, *n* = 3–6. The data were assessed by one‐way ANOVA and are shown as mean ± SEM. ^*^ *p* < 0.05, ^**^ *p* < 0.01, ^***^ *p* < 0.001.

Since we have demonstrated in vitro that MERTK modulates MMT via the SRC‐TKS5 pathway, we next construct a model of BLM‐induced pulmonary fibrosis in mice with macrophage‐specific knockout of MERTK to replicate this result. Consistent with the in vitro results, reduced expression of SPP1 and activity of SRC were found in lung tissues of mice with macrophage‐specific knockout of MERTK compared with littermate control mice (Figure 7A). In agreement with this, immunofluorescence staining also revealed suppression of TKS5 expression in macrophages in lung tissues of MERTK CKO mice (Figure 7B), suggesting that knockout of MERTK inhibits activation of the SRC‐TKS5 signaling in macrophages. Next, we examined the proportion of each type of macrophages in the lung tissues of each group of mice using flow cytometry, and found that knockout of MERTK decreased the proportion of M2‐polarized macrophages (Figure 7C), as well as the proportion of interstitial macrophages in the lung tissues (Figure 7D), and the proportion of macrophages in MMT was also reduced accordingly (Figure 7E), indicating that knockout of MERTK regulates the macrophage subpopulation and polarization status through the SRC‐TKS5 signaling pathway and thus affects MMT.

### 2.8 Knockdown of TKS5 Inhibits Macrophage M2 Polarization and MMT in Fibrotic Mice

**FIGURE 8:**
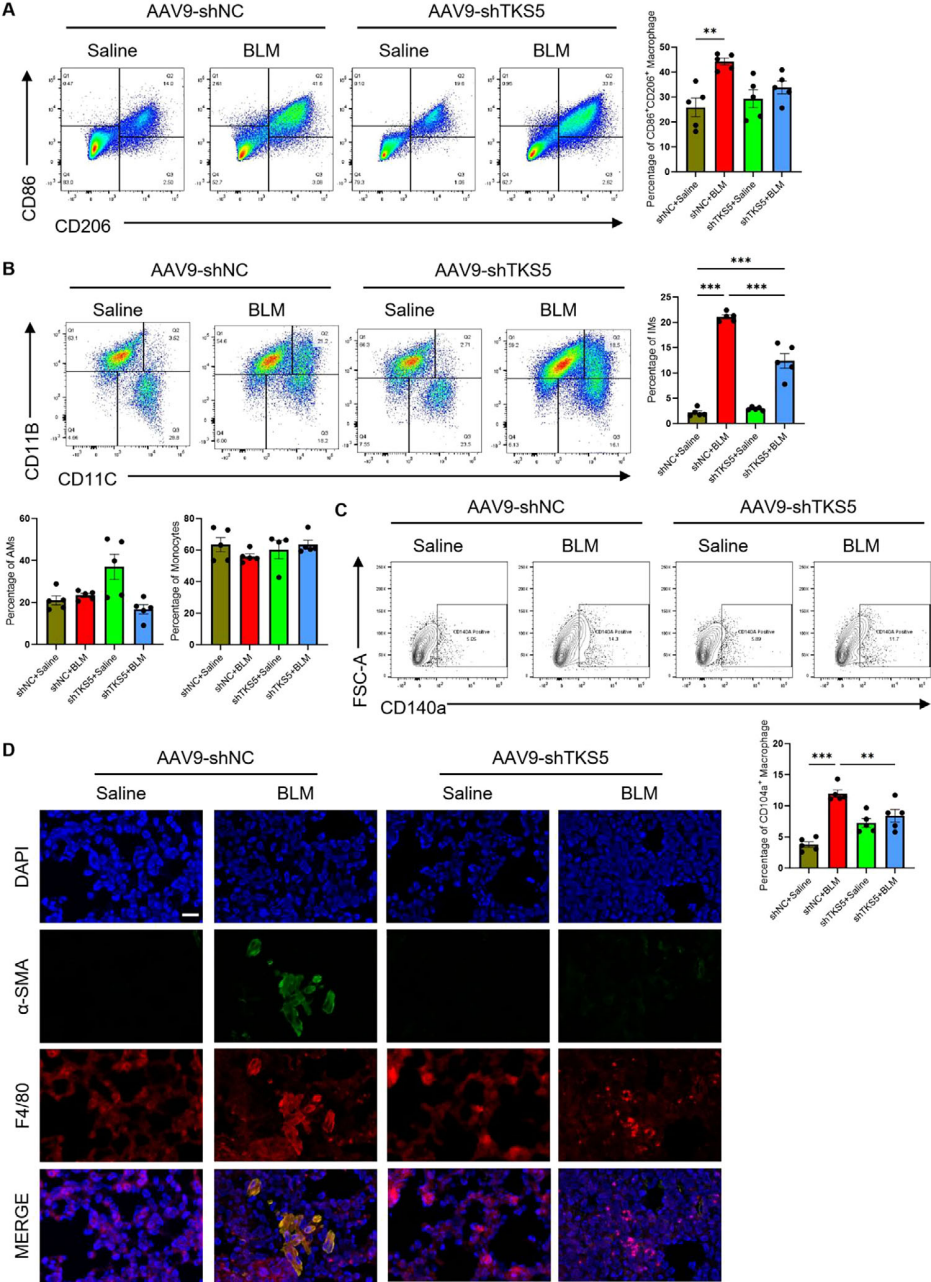
In vivo knockdown of TKS5 reduces macrophage M2 polarization and MMT. (A) Polarization of mouse lung macrophages in different groups detected by flow cytometry, *n* = 5. (B) Proportion of different types of macrophages in the lung tissue of each group of mice detected by flow cytometry, *n* = 5. (C) The ratio of MMT in macrophages from the lungs of mice in each group was detected by flow cytometry, *n* = 5. (D) Co‐localization of α‐SMA and F4/80 in lung tissues of mice from each group detected by immunofluorescence staining. The data were assessed by one‐way ANOVA and are shown as mean ± SEM. ^**^ *p* < 0.01, ^***^ *p* < 0.001.

After identifying the role of SRC‐TKS5 signaling in MMT, we then constructed TKS5 knockdown mice using AAV9 (Figure S5) to study the role of TKS5 in lung fibrosis. We first observed regulation of macrophage polarization by TKS5, and as shown in Figure 8A, TKS5 knockdown decreased the proportion of M2‐polarized macrophages (CD86^+^CD206^+^). Next, we examined the changes in the proportion of various subpopulations of macrophages in lung tissues, and found that knocking down TKS5 only reduced the proportion of interstitial macrophages, whereas the proportion of monocytes and alveolar macrophages remained unaffected (Figure 8B), suggesting that TKS5 may affect lung fibrosis mainly via regulating the infiltration of interstitial macrophages. Finally, we examined the percentage of cells that underwent MMT by flow cytometry, and the percentage of TKS5‐knockdown lung macrophages that sprouted MMT was markedly reduced (Figure 8C), as evidenced by the immunofluorescence staining results (Figure 8D). Collectively, these data demonstrate that TKS5 plays a regulatory role in macrophage subpopulations, polarization, and MMT.

### 2.9 Knockdown of TKS5 Inhibits BLM‐Induced Pulmonary Fibrosis in Mice

**FIGURE 9:**
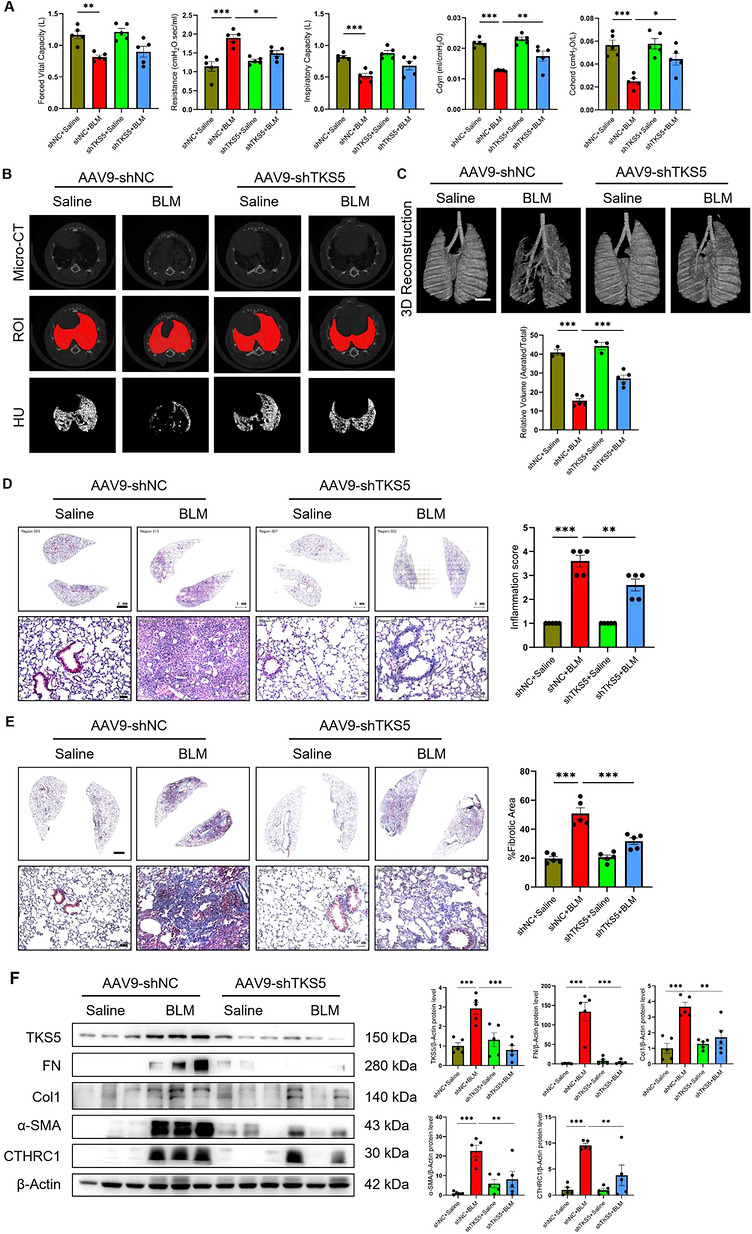
In vivo knockdown of TKS5 attenuates BLM‐induced pulmonary fibrosis. (A) Pulmonary function assay for different groups of mice, *n* = 5. (B) Detecting the extent of lung tissue damage in mice using microCT. (C) 3D reconstruction of mouse lung, and statistics on the proportion of aerated volume, scale bar = 2 mm, *n* = 3–5. (D) Hematoxylin‐Eosin (HE) staining was used to evaluate the degree of lung tissue injury in mice, scale bar = 1 mm (top) and 50 µm (bottom), *n* = 5. (E) Masson staining was used to assess the degree of fibrosis in mice, scale bar = 1 mm (top) and 50 µm (bottom), *n* = 5. (F) Western blot was used to detect the expression of fibrosis‐related proteins in lung samples from each group of mice, *n* = 5. The data were assessed by one‐way ANOVA and are shown as mean ± SEM. ^*^ *p* < 0.05, ^**^ *p* < 0.01, ^***^ *p* < 0.001.

Knockdown of TKS5 by AAV9 effectively attenuates BLM‐induced macrophage polarization abnormalities and reduces MMT, and we next investigated the effects of knocking down TKS5 on BLM‐induced lung fibrosis. TKS5 knockdown ameliorated BLM‐induced lung function impairment as evidenced by a reduction in lung tissue airway resistance (RI), dynamic compliance (Cdyn), static compliance (Cchord), forced lung volume (FVC), and deep inspiratory capacity (IC), as measured by lung function test (Figure 9A). Next, we imaged the mouse lung with microCT to comprehensively evaluate the extent of lung tissue damage, and found that BLM instillation induced remarkable changes in lung tissue, while TKS5 knockdown greatly improved lung tissue damage (Figure 9B). We visualized and analyzed the proportion of normally ventilated lung tissue areas using 3D reconstruction, and found that TKS5 knockdown enhanced the proportion of normally ventilated lung tissue in mice (Figure 9C), suggesting that lung tissue injury was alleviated. HE and Masson staining were used to assess the extent of inflammatory injury and fibrosis in mouse lung tissues, and it was found that TKS5 knockdown attenuated BLM‐induced inflammatory injury and fibrosis (Figure 9D,E). Finally, we examined the expression of fibrosis‐related molecules such as FN, Col1, α‐SMA, and CTHRC1 by WB. Increased expression of these proteins was found in the lungs of BLM‐instilled mice, whereas knockdown of TKS5 suppressed expression of these proteins (Figure 9F), suggesting that knockdown of TKS5 attenuates BLM‐induced lung fibrosis.

## 3 Discussion

Idiopathic pulmonary fibrosis, an interstitial lung disease with progressive pulmonary fibrosis, is characterized by excessive deposition of extracellular matrix arising from the main pathological feature [1, 2]. During fibrogenesis, fibroblast activation into myofibroblasts is the main source of extracellular matrix in the fibrotic microenvironment. However, it has also been shown that the origin of myofibroblasts in the fibrotic microenvironment is heterogeneous, and in addition to fibroblasts of traditional origin, epithelial, endothelial, and pericytes are also capable of differentiating into myofibroblasts with pro‐fibrotic properties through processes such as EMT (epithelial‐mesenchymal transition) [21], EndoMT (endothelial‐to‐mesenchymal transition) [22], and pericyte‐myofibroblast transformation (PMT) [23] to myofibroblasts with pro‐fibrotic properties. Macrophages are major players in innate immunity and participate in the pathogenesis of pulmonary fibrosis by modulating immune responses in the microenvironment [24]. Moreover, it has been found that macrophages can also drive the fibrotic process by replenishing myofibroblasts in the microenvironment via MMT during cardiac and renal fibrosis [25, 26]. In the present study, we found co‐localization of macrophage markers (CD68 or F4/80) with myofibroblast markers (α‐SMA) in the lung tissues of both IPF patients as well as BLM‐induced fibrotic mice, suggesting that MMT is present in fibrotic lung tissues. Further, we stimulated mouse‐derived and human‐derived macrophages with a pro‐fibrotic cytokine TGF‐β1, and obtained consistent results. These findings broaden our knowledge on the role of macrophages in IPF pathogenesis.

Macrophages maintain organ homeostasis by regulating immune responses. Our previous studies have demonstrated that altered macrophage function play important roles in pulmonary fibrosis [8, 27]. MMT, a term introduced in 2014 that refers to transformation of macrophages into myofibroblasts, contributes to organ fibrosis after injury and plays a significant role in the development and progression of fibrosis [28]. In recent years, MMT has been found in a variety of fibrosis‐related diseases [29, 30, 31]. In renal fibrosis, up to 50% of myofibroblasts are derived from macrophages, and the majority of MMT‐derived myofibroblasts express the macrophage M2 polarization marker CD206, suggesting that the occurrence of MMT in renal fibrosis is dependent on macrophage M2 polarization, and that M2‐type macrophages are the main cell of origin of MMT in renal fibrosis [32, 33]. MMT is also present in fibrotic lesions of the heart, retina, bladder, and other organs [30, 34, 35, 36], demonstrating its involvement during the fibrogenic process in a wide range of organs. Here, we found that lung macrophages have the potential to transdifferentiate into myofibroblasts with pro‐fibrotic capacity, as evidenced by an elevated expression of pro‐fibrotic marker CTHRC1. Furthermore, we found by flow cytometry that macrophages undergo predominantly M2 polarization in BLM‐induced pulmonary fibrosis, which is consistent with previous findings. Meanwhile, our analysis revealed that the macrophages undergoing MMT (CD140A‐positive macrophages) were predominantly M2 phenotype, and both bone marrow‐derived interstitial macrophages and tissue‐resident alveolar macrophages could undergo MMT. These findings suggest that MMT is involved in the development of pulmonary fibrosis.

The macrophage efferocytosis receptor MERTK (Mer tyrosine kinase) is one of the TAM (Tyro‐3, Axl, and Mer) family of tyrosine kinase receptors [37, 38], which promotes macrophage proliferation by mediating macrophage efferocytosis in apoptotic cells. [39] We previously demonstrated that MERTK is highly expressed in macrophages from patients and mice with pulmonary fibrosis, and it promotes lung fibrosis [8]; however, the enhanced pro‐fibrotic effect of MERTK in macrophages is not dependent on efferocytosis, and therefore, we hypothesized that MERTK may promote pulmonary fibrosis via other ways. In the present study, we found that macrophages undergoing MMT in fibrotic lung highly express MERTK and that activation of MERTK using GAS6, a ligand for MERTK, is sufficient to induce MMT in macrophages. Knockdown of MERTK in macrophages using siRNA decreases TGF‐β1‐induced MMT. Additionally, conditional knockout of MERTK in macrophages reduces MMT and attenuates BLM‐induced pulmonary fibrosis in mice. These evidences confirm that macrophage MERTK is elevated in lung fibrosis and it can promote lung fibrosis via inducing MMT.

Secreted phosphoprotein 1 (SPP1, also known as osteopontin) is a multifunctional glycoprotein belonging to the extracellular matrix protein family [40]. It is mainly expressed in activated macrophages and epithelial cells. SPP1 is highly expressed in fibrotic lungs and tightly linked to M2 polarization of macrophages. Inhibition of SPP1 attenuates BLM‐induced lung fibrosis [12]. MERTK regulates the expression, synthesis, and secretion of SPP1, and the transcription and activation of SPP1 mainly occur in MERTK^+^ M2‐type macrophages. In addition, inhibition of MERTK and macrophage M2‐type polarization leads to reduced SPP1 expression [41, 42]. A subpopulation of MERTK^+^SPP1^+^ macrophages was found to be highly proliferative in IPF lungs with a significant increase in cell number, and this subpopulation of cells is in close proximity to fibroblasts in fibrotic foci, [9] which may be involved in the fibrotic process of the lung tissue. In line with this, we found elevated expression of MERTK and SPP1 and highly co‐localized in fibrotic lung tissues, confirming the important role of MERTK^+^SPP1^+^ macrophages in lung fibrosis. MERTK activation enhanced expression of SPP1 while its knockdown decreased SPP1 expression in macrophages further confirmed the regulatory role of MERTK in SPP1, highlighting the importance of the interaction of these two proteins for the development of pulmonary fibrosis. Importantly, earlier single‑cell atlases and spatial studies established the existence and topographic enrichment of these MERTK^+^SPP1^+^ macrophages in human IPF, but did not demonstrate their capacity for transdifferentiation or identify the downstream molecular effectors. Our data extend those observations by showing that MERTK activation increases SPP1 expression and engages the SRC‐TKS5 signaling to promote MMT, and that targeting MERTK or TKS5 blocks this fate transition.

Increased invasive capacity of fibroblasts in the ECM is thought to be one of the main reasons for their recruitment during lung fibrosis, and the invasive capacity of fibroblasts is dependent on podosome formation [16]. Podosome consists of a filamentous‐actin‐rich (F‐actin‐rich) core enriched with actin‐regulatory proteins and is surrounded by a ring of scaffolding proteins, most notably the SH3 and PX structural domain 2A (SH3PXD2A, TKS5) [43]. Expression of TKS5 and formation of podosomes are important for fibroblast invasion in fibrotic environment [16]. Moreover, TKS5 is also able to regulate macrophage migration and invasive capacity, affecting macrophage podosome formation and cell fusion [15]. TKS5 activity is dependent on SRC tyrosine kinase‐mediated phosphorylation [44], and this activation process is regulated by SPP1 [14]. Our experiments revealed that the SRC‐TKS5 pathway was regulated by MERTK, and TKS5 expression was increased in fibrotic macrophages. Activation of MERTK promotes SRC activation and TKS5 expression in macrophages, whereas knockdown of MERTK inhibits the activity of SRC‐TKS5. Further, adeno‐associated virus (AAV)‐mediated knockdown of TKS5 in the lung reduced the degree of BLM‐induced MMT and pulmonary fibrosis, substantially improving lung function. These interventional experiments provide causal evidence‐rather than correlative association‐that the SRC‐TKS5 module, downstream of MERTK‐SPP1, is necessary for MMT and fibrotic progression.

In summary, we found that macrophages undergo MMT during pulmonary fibrosis and that the myofibroblasts transdifferentiated from macrophages exhibit pro‐fibrotic properties. MMT is dependent on the MERTK‐SPP1‐SRC‐TKS5 signaling axis, and activation of MERTK induces MMT in macrophages through upregulation of SRC‐TKS5 activity by SPP1. Specific knockout of MERTK in macrophages reduces SRC‐TKS5 activity, which reduces MMT and ultimately attenuates lung fibrosis. At the same time, knockdown of TKS5 also inhibits MMT and attenuates lung fibrosis. We found that targeted inhibition of the MERTK‐SPP1‐SRC‐TKS5 signaling axis effectively attenuates pulmonary fibrosis and decreases lung function decline, offering important translational implications for the treatment of pulmonary fibrosis.

## 4 Materials and Methods

### 4.1 Human Samples

The study protocol was approved by the Ethics Committee of the First Affiliated Hospital of Guangzhou Medical University (Approval No. 2018–92), and written informed consent was obtained from each subject. Patients with IPF were included in the study according to the diagnostic criteria of the 2018 ATS/ERS/JRS/ALAT clinical practice guidelines [45]. Lung tissues from IPF subjects or healthy controls were collected for verification of MMT and MERTK expression.

### 4.2 Animal Experiments

All animal experiments were approved by the Ethics Committee of The First Affiliated Hospital of Guangzhou Medical University (Approval No. 20240649). The mouse model of pulmonary fibrosis was established in accordance with our previously published research [46]. Male C57BL/6J mice (8 weeks old) were purchased from Hua FuKang Co., Ltd. (Beijing, China). All mice were given free access to water and food and were housed in the Specific Pathogen Free laboratory animal center of Guangzhou Medical University. To induce lung fibrosis, mice were intratracheally administered 50 µL BLM solution after anesthesia. Mice in the control group were administered an equal volume of sterile saline. To investigate the role of MERTK in MMT and pulmonary fibrosis, MERTK‐flox mice (Strain S‐CKO‐03713) and Lyz2‐ Cre mice (Catalog C001358) were purchased from Cyagen Biosciences, Inc. to generate MERTK^flox/flox^; Lyz2^Cre^ mice for the construction of the BLM‐induced pulmonary fibrosis model, and their Littermates were used as control mice. To investigate the role of TKS5 in MMT and lung fibrosis, shTKS5 AAV9 was purchased from PackGene Biotech Inc. to knock down TKS5 in mice, followed by intratracheal administration of BLM to induce lung fibrosis.

### 4.3 Pulmonary Function Measurements

Pulmonary function was assessed with the Forced Pulmonary Maneuver System (Buxco Research Systems, USA) following the manufacturer's protocol. Indicators such as forced vital capacity, resistance, and cdyn were used to analyze pulmonary function in mice.

### 4.4 MicroCT Analysis

Mice were anesthetized and transferred to microCT (Skyscan 1276; Bruker microCT) for high‐resolution scanning according to the manufacturer's instructions. The data were denoised using CT Analyzer 1.16.4.1, and the ratio of normally aerated area to total lung volume was quantified. 3D reconstruction and rendering were performed using CTvox 3.3 software.

### 4.5 Histologic Analysis and Evaluation of Fibrosis Degree

Hematoxylin & Eosin (HE) staining was used to assess the inflammatory infiltration of lung tissue, and Masson's trichrome was used to assess the degree of fibrosis of lung tissue.

### 4.6 Western Blot Analysis

Lung tissue or cellular protein samples were prepared with RIPA lysate, and protein quantification was performed using the BCA Protein Analysis Kit. Equal amounts of proteins were separated by SDS‐PAGE and transferred to PVDF membrane, blocked with 5% skimmed milk for 1 h, and incubated with the indicated antibodies overnight. The secondary antibody was added for 1 h, and the detection was performed using Tanon 5200 (Tanon, Shanghai, China). All primary antibodies are listed in Table S1.

### 4.7 Cell Staining and Flow Cytometry

Samples for flow cytometry staining were prepared according to guidelines. Before staining, cells were suspended in anti‐rat Fc receptor (CD16/32) for blocking, followed by surface staining according to different panels. FACS Verse (BD Bioscience, USA) was used to perform flow cytometry assays. FACS data were analyzed using FlowJo 10.8 software. The antibodies used in this study are listed in Table S2.

### 4.8 Cell Culture and Treatment

Mouse‐derived MH‐S cells and human‐derived THP‐1 cells were obtained from ATCC. Cells were cultured in 1640 medium containing 10% FBS, 100 U/mL penicillin, and 100 µg/mL streptomycin. THP‐1 cells were induced to differentiate into macrophages using PMA before subsequent experiments. Transfected or untransfected cells were stimulated with TGF‐β1 or GAS6 for 48 h, depending on experimental requirements.

### 4.9 Immunofluorescence Staining

Cells were fixed with 4% paraformaldehyde, permeabilized in 0.02% Triton X‐100, and blocked with goat serum. Primary antibodies were diluted with blocking buffer and incubated overnight at 4°C. FITC‐conjugated or TRITC‐conjugated secondary antibodies were added for 1 h. Nuclei were counterstained with DAPI‐containing mounting solution. When performing immunofluorescence staining of lung tissue, paraffin sections need to be pre‐deparaffinized and antigen retrieval with sodium citrate. Results were visualized by fluorescence microscopy. Results were analyzed by ImageJ.

### 4.10 Statistical Analyses

Data were graphed using GraphPad Prism 10 software (Graph Pad Software Inc.) and are expressed as mean ± SEM. Comparisons between two groups were analyzed using unpaired two‐sided Student's *t*‐tests, and comparisons between multiple groups (≥3 groups) were analyzed using one‐way analysis of variance (ANOVA). *p*‐values less than 0.05 were considered statistically significant.

## Author Contributions

X.X.T. conceived, designed, and supervised the study. Y.W., H.G., and X.Y. conducted experiments. X.X.T. and Y.W. interpreted the data and wrote the manuscript. All authors have read and approved the manuscript.

## Funding

This study was supported by the National Natural Science Foundation of China (82470065 and 82270077), R&D Program of Guangzhou National Laboratory (GZNL2025A02002), Project GZNL2025B01005 supported by Guangzhou National Laboratory and State Key Laboratory of Respiratory Disease (GZNL2025B01005), National Science and Technology Major Project for Innovative Drug Development (SQ2026AAA161178), and Guangzhou Institute of Respiratory Health Open Project.

## Conflicts of Interest

The authors declare no conflicts of interest.

## Supporting information




**Supporting File**: advs75620‐sup‐0001‐SuppMat.pdf.

## Data Availability

Data sharing not applicable to this article as no datasets were generated or analysed during the current study.
