# Supplementary material for: Macrophage‐to‐Myofibroblast Transdifferentiation Contributes to Pulmonary Fibrosis via the MERTK‐SPP1‐SRC‐TKS5 Signaling Axis
Source: Adv Sci (Weinh). 2026 May 14;13(47):e75620. doi: 10.1002/advs.75620 (PMC13336014; doi:10.1002/advs.75620)
Supplement: Supplementary file 1 [file ADVS-13-e75620-s001.pdf]

ONLINE DATA SUPPLEMENT

Supplementary Tables

Table S1. List of antibodies used for WB analysis

Target	Cat. No.
MERTK	ab300136, Abcam
SRC	ab47405, Abcam
p-SRC	ab185617, Abcam
TKS5	18976-1-AP, Proteintech
SPP1	Ab63856, Abcam
FN	sc-8422, Santa Cruz
Col-I	14695-1-AP, Proteintech
α-SMA	ab5694, Abcam
β-Actin	8457, CST

Table S2. List of anti-mouse antibodies used for flow cytometry

Target	Conjugate	Cat. No.
CD45	APC/Cy7	30-F11, Biolegend
CD11b	Percp/Cy5.5	M1/70, BD Bioscience
CD11c	PE/Cy7	HL3, BD Bioscience
F4/80	PE	BM8, Biolegend
F4/80	BV421	T45-2342 (RUO), BD Bioscience
CD140a	APC	APA5, Biolegend
MERTK	PE/Cy7	2B10C42, Biolegend
CD206	BV421	C068C2, Biolegend
CD86	BV510	GL-1, Biolegend

Fig. S1 Macrophages undergoing MMT in pulmonary fibrosis are polarized toward M2 phenotype.

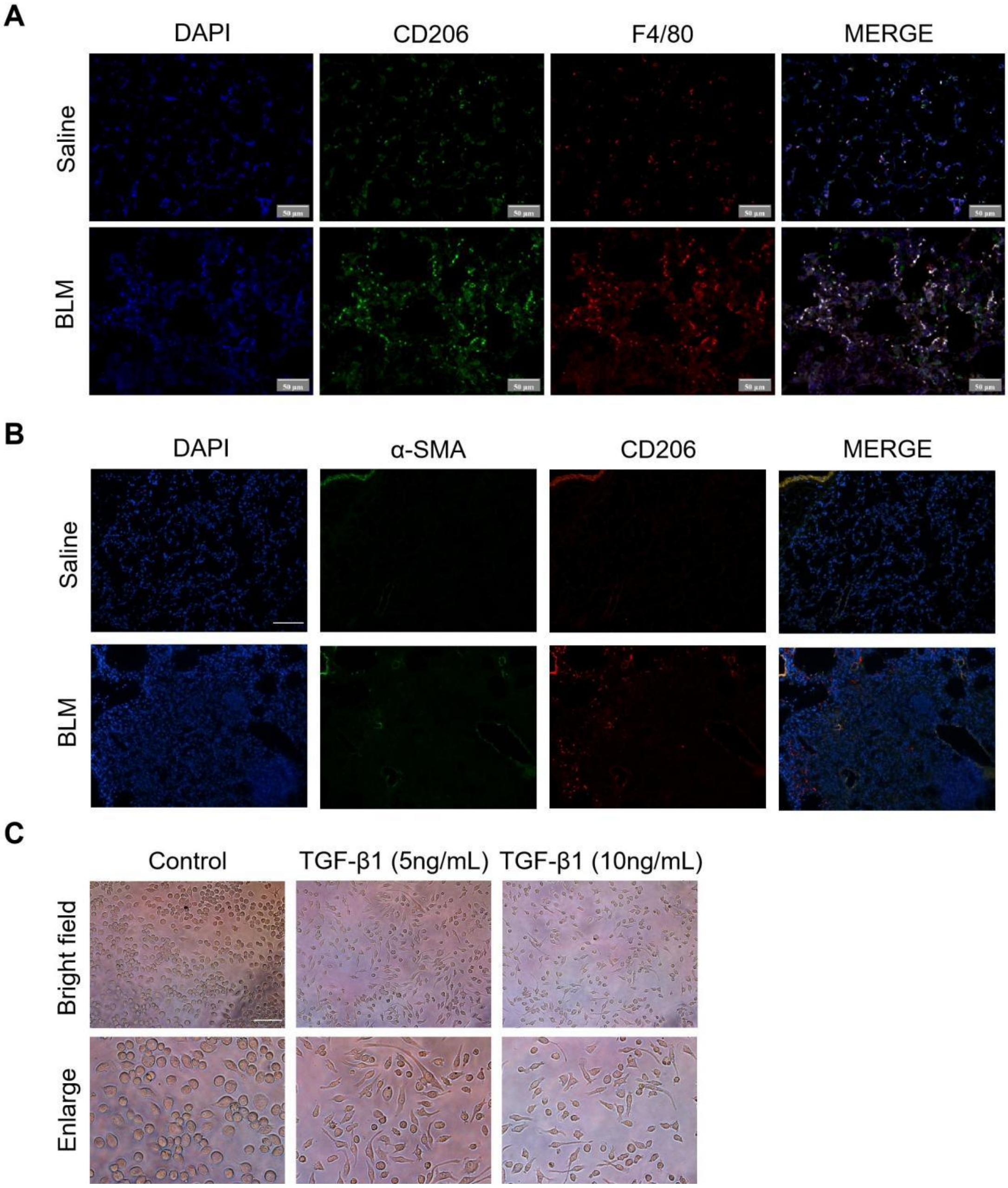

A. Detection of macrophage M2 polarization in lung tissue sections of bleomycin-induced lung fibrotic mice by immunofluorescence staining. Scale bar=50 μm.

B. Immunofluorescence staining was used to detect co-localization of the fibroblast marker α-SMA with the macrophage M2-type marker CD206 in lung tissue sections of bleomycin-induced lung fibrotic mice. Scale bar=20 μm.

C. Morphological changes including cell elongation and spindle-like changes in

TGF-β1-induced MH-S cells were observed under the microscope.

Fig. S2 Primary cultured macrophages undergo MMT.

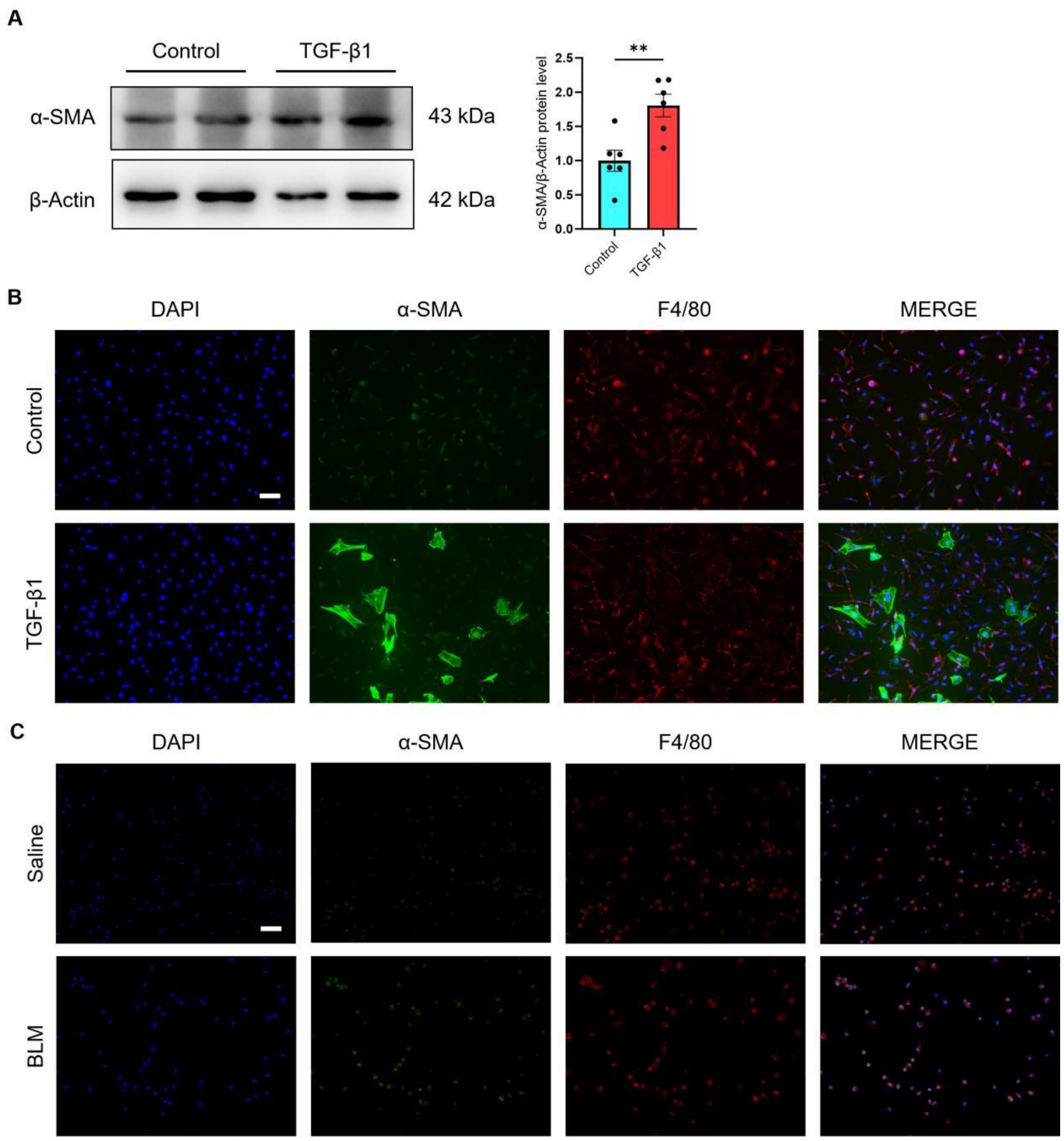

A. Mouse bone marrow cells were isolated and induced to differentiate into BMDMs using 100 ng/mL MCSF; they were then treated with 10 ng/mL TGF-β1 for 48 hours, and proteins were extracted to detect α-SMA expression, n = 6.

B. After BMDM cells were stimulated with TGF-β1 for 48 hours, α-SMA expression was detected by immunofluorescence staining. Scale bar = 20 μm.

C. Bronchoalveolar lavage fluid (BALF) was collected from the mice of Saline group

and BLM group, and alveolar macrophages were isolated from BALF; immunofluorescence staining was performed to detect α-SMA expression. Scale bar = 20 μm.

Fig. S3 TGF-β1 enhances expression of MERTK and SPP1 in macrophages.

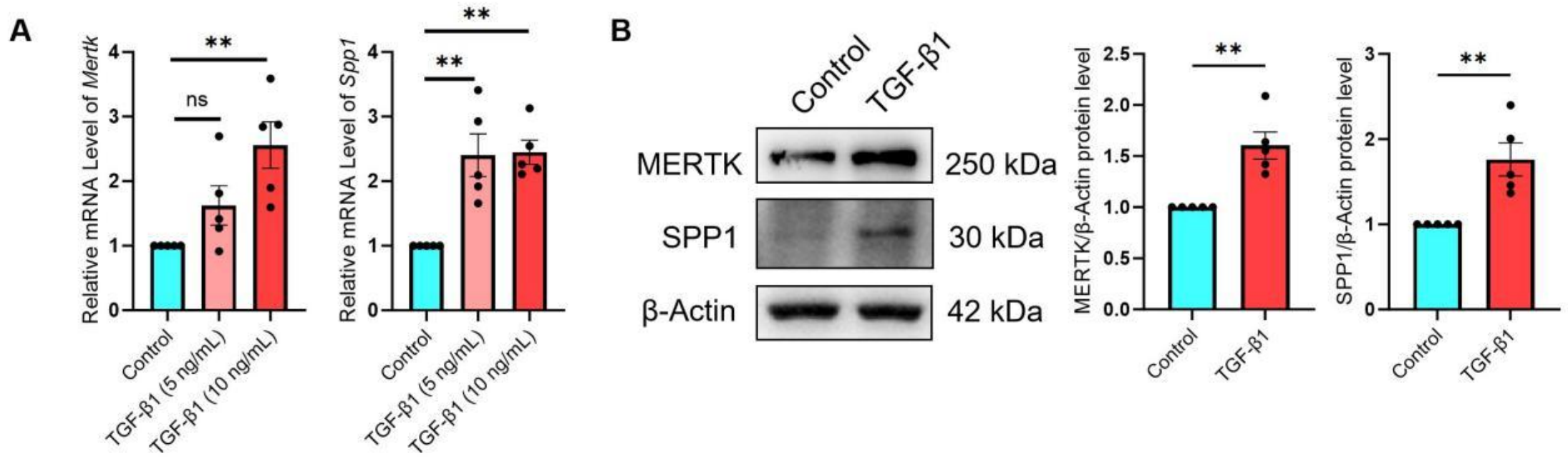

A. qPCR detection of MERTK and SPP1 gene expression in TGF-β1-induced MH-S cells, n=5. The data were assessed by one-way ANOVA and are shown as mean ± SEM. ns means not significant, $^{**}P < 0.01$.

B. WB detection of MERTK and SPP1 expression in TGF-β1-induced MH-S cells, n=5. The data were assessed by unpaired two-sided Student's t-test and are shown as mean ± SEM. $^{**}P < 0.01$.

Fig. S4 Depiction of the cross used to generate myeloid-specific Mertk knockout mice.

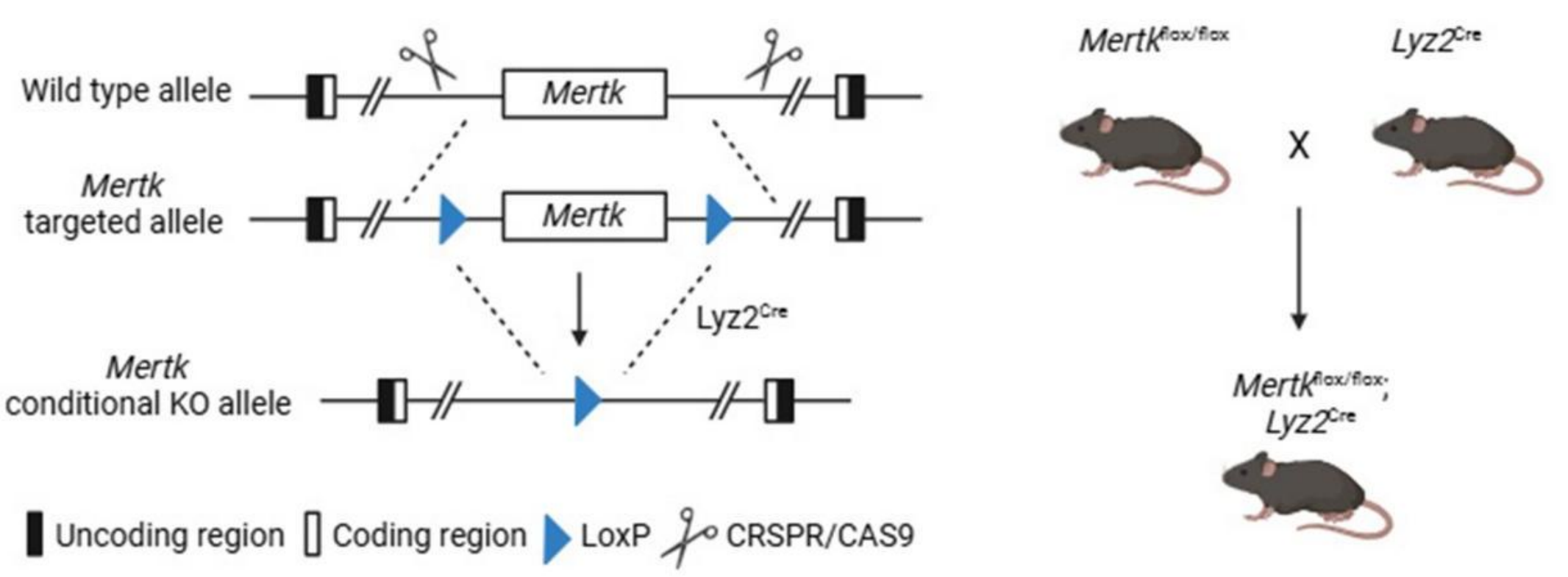

Mice carrying Lyz2-Cre transgene (Catalog C001358) were crossed with Mertk-floxed (Strain S-CKO-03713) mice to obtain Mertkflox/flox; Lyz2+/+ (control) and Mertkflox/flox; Lyz2Cre (Mertk CKO) mice.

Fig. S5 Validation of AAV-mediated TKS5 knockdown efficiency.

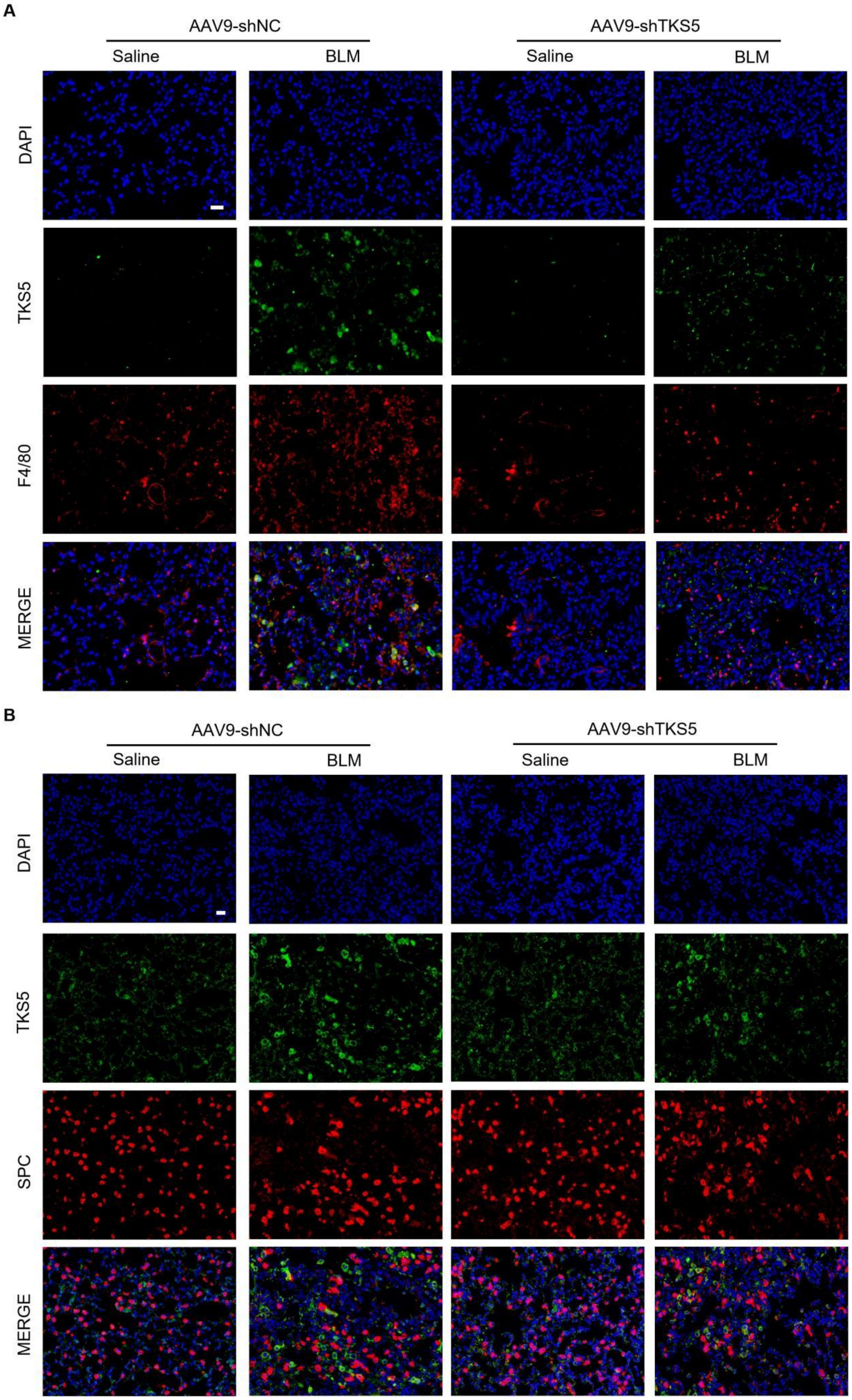
A
AAV9-shNC
AAV9-shTKS5
Saline
BLM
Saline
BLM
DAPI
TKS5
F4/80
MERGE
B
AAV9-shNC
AAV9-shTKS5
Saline
BLM
Saline
BLM
DAPI
TKS5
SPC
MERGE

A. The efficiency of TKS5 knockdown in lung tissue macrophages by adeno-associated virus (AAV) was assessed via immunofluorescence staining. Co-localization of the macrophage marker F4/80 with TKS5 was examined. AAV9-shTKS5 effectively knocked down BLM-induced TKS5 expression in macrophages. Scale bar = 20 μm.

B. Immunofluorescence staining was performed to evaluate the efficiency of AAV-mediated TKS5 knockdown in lung tissue epithelial cells and to examine the co-localization of TKS5 with the epithelial cell marker SPC. Although AAV9-shTKS5 effectively knocked down BLM-induced TKS5 expression, TKS5 was not predominantly expressed in epithelial cells. Scale bar = 20 μm.
